# Fragmentation Patterns of Phenolic C-Glycosides in Mass Spectrometry Analysis

**DOI:** 10.3390/molecules29132953

**Published:** 2024-06-21

**Authors:** Ting Du, Yang Wang, Huan Xie, Dong Liang, Song Gao

**Affiliations:** Department of Pharmaceutical Science, College of Pharmacy and Health Sciences, Texas Southern University, 3100 Cleburne Street, Houston, TX 77004, USA; du.ting@tsu.edu (T.D.); yang.wang@tsu.edu (Y.W.);

**Keywords:** phenolic C-glycosides, MS/MS, fragmentation pattern, UPLC-QTOF-MS

## Abstract

Background: Many phenolic C-glycosides possess nutritional benefits and pharmacological efficacies. However, the MS/MS fragmentation pattern of phenolic C-glycosides analysis is understudied. This paper aims to determine the MS/MS fragmentation patterns of phenolic C-glycosides. Method: Ten compounds with different sugar moieties, aglycones, and substitutes were analyzed to determine the impact of these structural features on MS/MS fragmentation using UPLC-QTOF-MS analysis. Results: The results showed that water loss followed by RDA reaction and alpha cleavage in the C-C bonded sugar moieties are the major fragmentation pathways. Additionally, the sugar cleavage was not affected by the skeleton and the substitute of the aglycones. These results suggested that the fragmentation patterns of phenolic C-glycosides differ from those in the O-glycosides, where the O-C glycosidic bond is the most cleavage-liable bond in MS/MS analysis. Conclusions: These MS/MS fragmentation patterns can be used for the identification of C-glycosides from dietary components and herbal medicine as well as developing robust methods using MRM methods to quantify C-glycosides.

## 1. Introduction

C-glycosides, a class of compounds in which one or more sugar moieties is/are attached to an aglycone via a carbon–carbon (C-C) bond, are widely distributed in various of fruits, vegetables, and herbal materials. It was reported that more than 750 c-glycosides have been identified from plants and many of the aglycones of these identified glycosides are phenolic compounds [1,2]. The biosynthesis of C-glycosides is mediated using C-glycosyltransferases (CGTs) and there are at least 66 CGTs isoforms have been identified in plants. Several recent review articles have summarized the structures, bioactivates, and chemical and biosynthesis of C-glycosides [1,3,4,5].

In addition to nutritional benefits, many dietary C-glycosides possess pharmacological effects, including anti-inflammatory, cardioprotective, antioxidant, anti-cancer, antidiabetic, neuroprotective effects, hepatoprotective, and immunomodulatory properties [5]. Therefore, dietary C-glycosides have been gaining increasing attention from pharmacologists and some of these compounds have been subjected to preclinical and clinical studies. For example, mangiferin, a C-glycoside isolated from mango fruit, has been found to possess cancer prevention, immunomodulation, serum lipid profile improvement, and antidiabetic effects in preclinical and clinical studies [6]. Mechanism studies revealed that the bioactivity of C-glycosides is mainly from the aglycones. Whereas pharmaceutical studies demonstrated that the sugar moiety in the structures can improve water solubility, making the glycoside forms more favorable in pharmaceutical and pharmacological studies [7].

C-glycosides are generally considered to be more stable than O-glycosides towards glycoside hydrolases (also called glycosidases or glycosyl hydrolases) in mammalian cells [8]. Therefore, these glycosides theoretically can be absorbed without being hydrolyzed and may be the compounds responsible for the biological activity. On the contrary, it has been reported that the C-C bond in C-glycosides could be cleaved by intestinal microbes to release the aglycone for various bioactivities in vivo [9]. This debate highlights the importance of bioanalysis of C-glycosides and their metabolites in pharmaceutical studies. 

The liquid chromatography-mass spectrometry (LC-MS) technique is widely used in pharmaceutical and chemistry studies for the identification of phenolic O-glycosides. This is because the sugar type and connection position, particularly when multiple sugars are present with one or more sugar chains, can be readily identified using MS/MS, neutral loss, or precursor scans. Moreover, the glycosidic bond in O-glycosides (i.e., C-O bond) is the easiest bond to be cleaved in MS/MS analysis and the adduct ions from sugar moiety loss are often used in the MRM scan for quantification [10]. However, in C-glycoside, the sugar moiety is attached with the aglycone via a C-C bond, which may not be the easiest bond to be cleaved in MS/MS analysis [11]. Previous studies have reported that sugar moiety loss is not the most common fragmentation pathway in C-glycosides as it is in O-glycoside rather than the cleavage in the sugar moiety [11,12,13,14]. However, it is still understudied whether the cleavage in the sugar moiety is affected by the structure of the compounds. 

In this paper, we aimed to determine the fragmentation patterns of C-glycosides using ten dietary phenolic C-glycosides with UPLC-QTOF-MS/MS. These compounds have different skeletons and substitutes, enabling us to determine the potential impact of aglycone on MS/MS fragmentation. Additionally, these compounds have various sugar types, numbers, sugar chains, and positions, allowing us to determine the potential impact of sugar moiety/chains on the fragmentation pathways. We anticipate that the identified fragmentation pathways can be used to elucidate the structure of phenolic C-glycosides from herbal products and to develop sensitive and robust triple Quadrupole MS quantification methods with MRM scan for the quality control of herbal products.

## 2. Results

### 2.1. Phenolic C-Glycoside Selection

To investigate the MS/MS patterns of dietary phenolic C-glycosides, we selected ten compounds with different structures, as shown in Table 1. The primary dietary sources of these compounds are also in Table 1. Among these compounds, vitexin, isovitexin, homoorientin, isoscoparin, and swertisin are flavonoids with sugar moiety(ies) at different positions, with hydroxyl and methoxys as the substitute(s) on the aglycones. By analyzing the MS/MS patterns of these compounds, we can determine the potential impact of the sugar position and the type/position of the substitutes on the fragmentation pathways. Another compound aloesin has a C-glucose moiety in the structure, but the lack of the B ring in the aglycone allows us to determine the potential impact of the B ring in flavonoids on C-glycoside cleavage in MS/MS analysis. Isoshaftoside has a glucose and a xylose moiety connected to the flavonoid aglycone via a C-C bond, while isoscoparin-2″-O-glucopyranoside has one sugar chain with a C-O glucose attached to the C2″ of the first glucose, which in turn attaches to the aglycone via a C-C bond. These two compounds will enable us to compare the MS fragmentation pathways of C-glycosides and O-glycosides. Mangiferin and neomangiferin both have the same xanthone skeleton as the aglycone. However, mangiferin has a C-glucose moiety attached to its aglycone, while neomangiferin has a C-glucose and an O-glucose moiety. This difference in structure will enable us to investigate the potential impact of aglycone structures on MS/MS patterns when compared with those glycosides with flavonoids as their aglycone. Additionally, the C- and O-glycoside moieties in these two xanthones will allow us to further compare the fragmentation behaviors of C- and O-glycosides. Table 2 lists the fragmentation pathways, the fragment ions, along with their intensities of each compound. 

### 2.2. Fragmentation Patterns of Dietary Phenolic C-Glycosides

#### 2.2.1. Glycosidic Bond Cleavage

**Figure 1:**
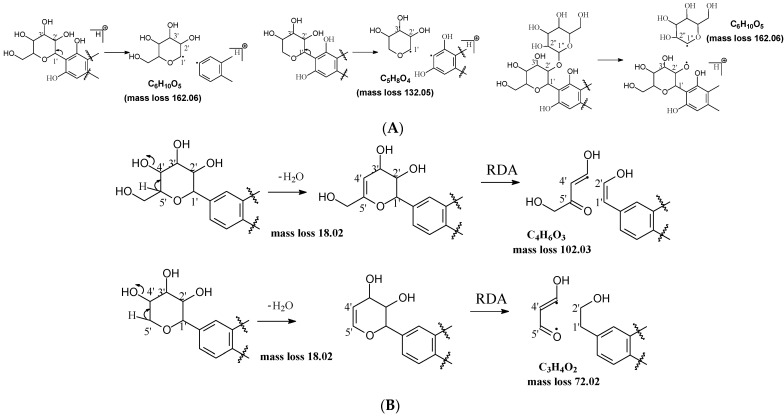

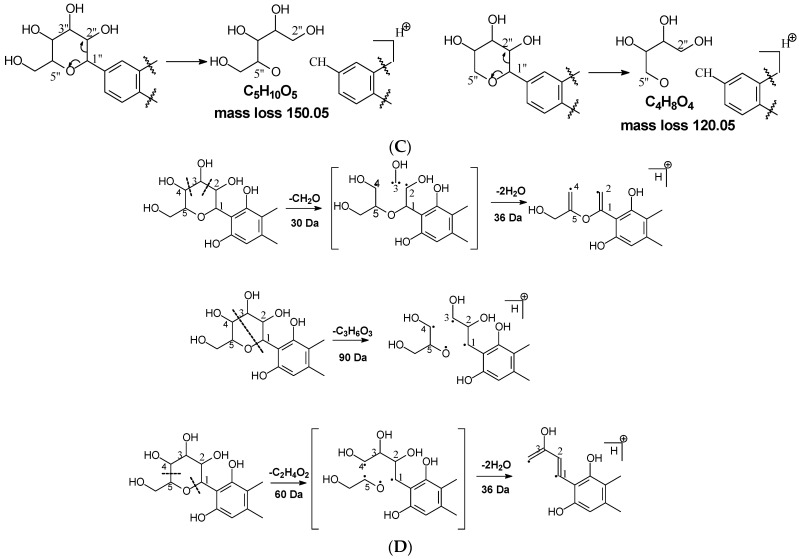
Fragmentation pathway of glycosidic bonds cleavage. (**A**), glycosidic bond cleavage; (**B**), water loss followed by RDA cleavage; (**C**), alpha cleavage; (**D**), other sugar cleavage.

**Figure 2:**
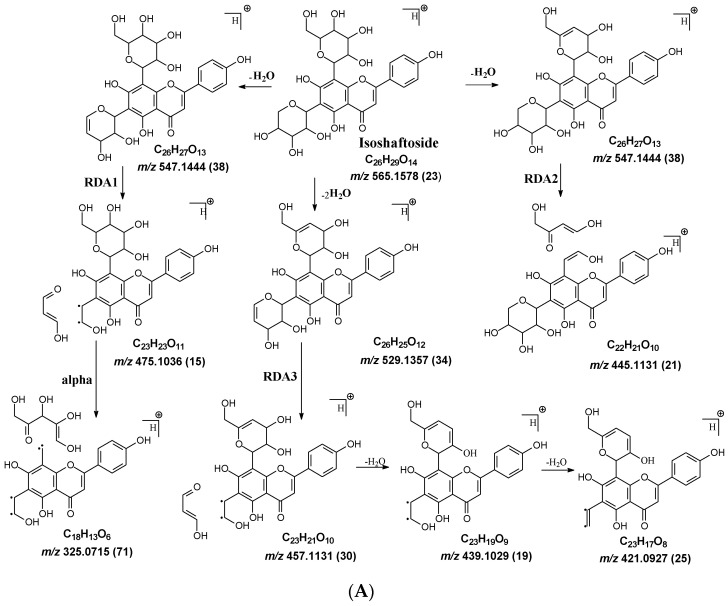

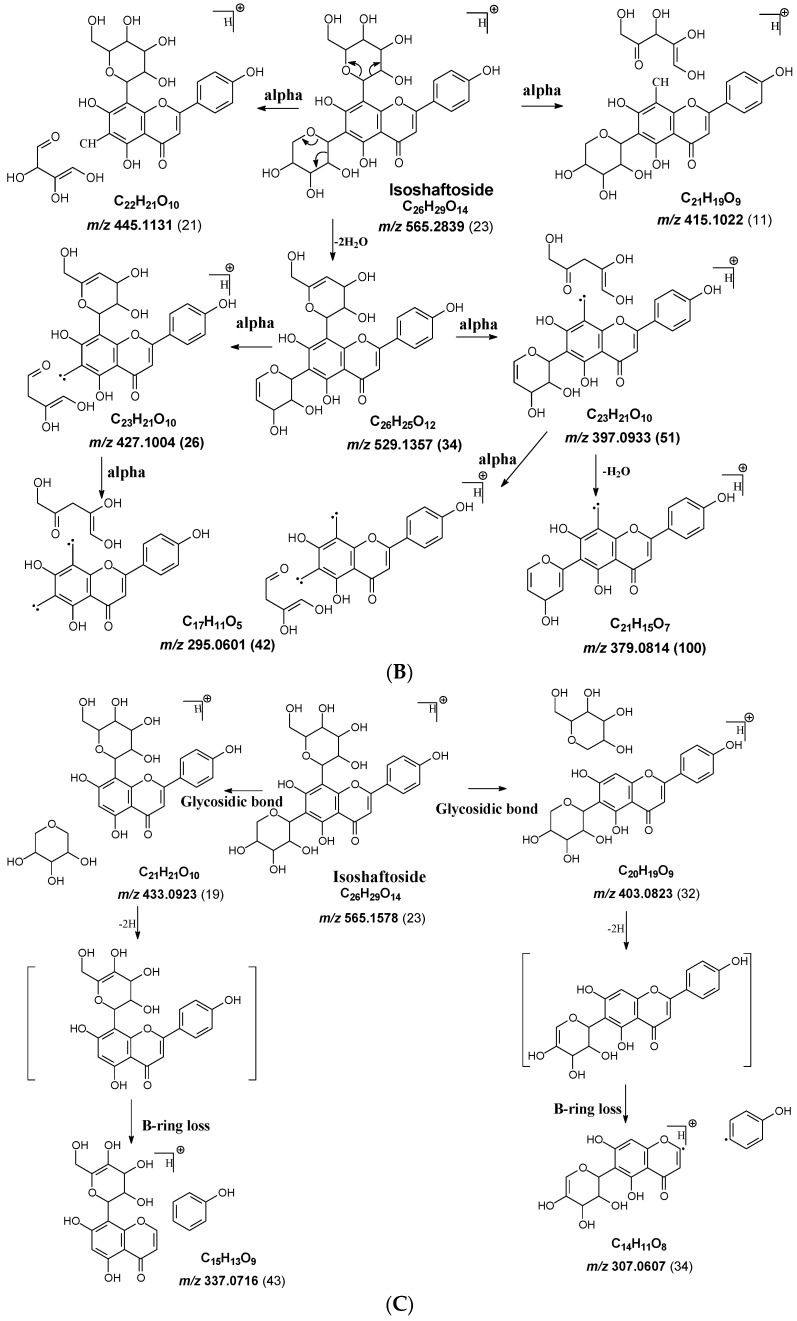

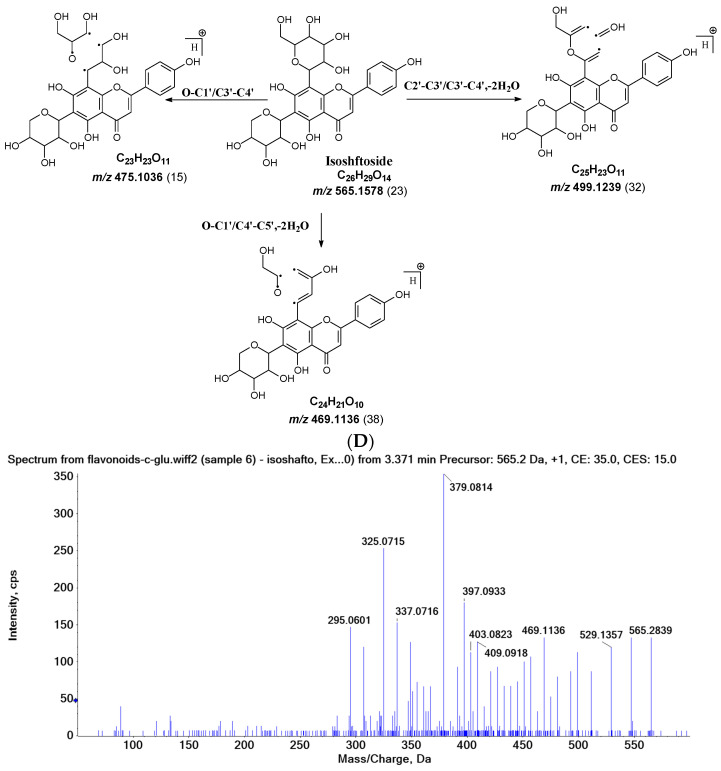
Fragmentation pathway of isoshaftoside. (**A**), water loss accomplished with RDA in the sugar moieties; (**B**), alpha cleavage in the sugar moieties; (**C**), glycosidic bond cleavage accomplished with B ring loss; (**D**), other types of sugar cleavage.

C-C and C-O glycosidic bond cleavage: In C-glycosides, the C-C glycosidic bond can be cleaved to afford a fragment ion losing 162 for glucose or 132 for xylose from the parents adduct ion in MS/MS analysis, which is similar to that in O-glycoside [11]. However, the C-C glycosidic bond cleavage is not the major fragment pathway as the abundance of the relevant fragment is low, except for aloesin, which releases the aglycone at *m*/*z* 233.0815 with 90% abundance (Figure 1A, Table 2). In isoshaftoside, both the glucose and the xylose moiety can be cleaved from the aglycone to afford fragment ions at *m*/*z* 403.0823 (32%) and *m*/*z* at 433.0932 (19%), respectively (Table 2, Figure 2), suggesting that the sugar type has little impact on C-C glycosidic bond cleavage [11]. In isoscoparin-2″-O-glucoside (Supplement Figure S6), the major fragment ion is the one that loses the O-glucose from the 2″ position on the C-glucose, with *m*/*z* 463.1231 and an abundance of 75% [26]. Similarly, in neomangiferin, the fragment ion from losing the O-glucose with the addition of a water unit at *m*/*z* 405.0812 (26%) is higher than that of losing C-glucose (Table 2) [27]. These findings suggest that the O-glycosidic bond is easier to be cleaved when compared to that of the C-glycosidic bond. This is probably because the O is easy to be ionized and O-C bond is easier to be cleaved compared to the C-C bond under the conditions of MS analysis.

#### 2.2.2. Sugar Cleavage

*Water loss followed retro-Diels-Alde cleavage (Water-loss-RDA) in the C-sugar moiety*: Water loss followed by RDA cleavage in the C-sugar moiety is the major fragmentation pathway for all these C-glycosides. In the C-C bonded sugar moiety, the water loss is suspected at C4′ and C5′ to form a double bond between C4′ and C5′ to afford a six-member ring pseudo-sugar moiety fragment (Figure 1B) with a loss of 18 Da for all the analytes (Table 2). This six-member ring sugar moiety can be further cleaved from O–C1″ and C2″–C3″ to form an RDA cleavage with a loss of 102 Da in glucose or 72 Da in xylose (Figure 1B). The abundance of the fragments from water loss followed by RDA fragmentation are relatively high in all these analytes, suggesting that these are the major fragments that can be used for quantification using MRM approach. Additionally, the relatively high abundance of water loss followed by RDA cleavage fragments in all these compounds further suggested that this fragmentation pathway is neither affected by the skeleton and substitutes of the aglycone nor by the sugar type and position in C–glycosides. This water loss incorporated with sugar RDA cleavage is not observed in the O-glucose moiety in isoscoparin-2′′-O-glucoside and neomangiferin, indicating that the chance of sugar cleavage in O-glycosides is low, probably because the O–glycosidic bond is more liable to be broken when compared to C-C bond in the sugar moiety.

In isoscoparin-2′′–O–glucoside, the base peak in MS/MS is *m*/*z* 343.0812 (100%), which is from the fragments with a loss of the O–glucose from the sugar chain followed by water-RDA cleavage on the C-glucose moiety. This result further suggests that O-glycoside is more cleavage-liable than that of C-glycoside. Interestingly, in neomangiferin, where there is a C-glucose and an O-glucose attached at different position on the aglycone, a fragment ion *m*/*z* 465.1028 (36%) from water-RDA cleavage in the C-glucose is observed.

These findings imply that water-RDA cleavage in C-glycoses can be cleaved without losing the O-glycoside if there is any in the structure.

In addition to water loss followed by RDA cleavage, double or triple water loss in the C-glycoside sugar moiety was also observed in all these analytes, resulting in fragment ions with a loss 36 or 54 Da, respectively (Table 2). The intensity of these double or triple water loss fragment ions ranges from low to medium. When there is an O-glucoside in the structures (i.e., isoscoparin-2″–O–glucoside, neomangiferin), double and triple water loss in the C-glycoside sugar occurs after the loss of the O-glucose.

*Alpha cleavage in the C-sugar moiety*: Cleavage from the alpha position of the O atom in the C-glycoside sugar is another major fragmentation pathway in MS/MS analysis. The cleavage occurs at the O–C1″and C1″–C2″ position to lose 150 Da for glucoside or 120 Da for xylose (Figure 1C). The abundance of this alpha cleavage is the base fragment ion with an abundance of 100% for isovitexin (Supplement Figure S1), homoorientin (Supplement Figure S2), isoscoparin (Supplement Figure S3), swertisin (Supplement Figure S4), and vitexin (Supplement Figure S5), in which the sugar moiety is attached to the aglycone at C6 (Table 1). These discovered compounds have different substitutes (i.e., −OH, or −OCH3) at different positions, suggesting that these substitutes have little impact on the sugar alpha cleavage. However, when the sugar is attached at position-8 on the aglycone in isovitexin, the intensity of the fragment ion from this sugar alpha cleavage is only 16% (Table 2), suggesting that the sugar position may affect the alpha cleavage. For isoshaftoside, in which there are a glucose at C-8 and a xylose at C-6, alpha cleavages were observed in both the glucose and xylose moieties to afford two fragment ions at *m*/*z* 445.1131 and 415.1022 with an abundance of 21% or 11%, respectively (Figure 2, Table 2). These findings suggested that sugar alpha cleavage can occur in different sugar types at different positions.

*Other cleavages in the C-sugar moiety*: The C-sugar moiety can also cleave from C2″–C3″ and C3″–C4″ and then lose two H_2_O to form a fragment with a loss of 30 + 36 Da, cleave from O–C1″ and C3″–C4″ to lose 90 Da, and from O–C1″ and C4″–C5″, losing two H_2_O to form a fragment ion with a loss of 60 + 36 Da (Figure 1D). These sugar cleavages only occur in C-glycoside in these analytes and the abundance of the relevant fragments ranges from low to medium (Table 2). When there is an O-glucose in the structure of isoscoparin-2″-O-glucopyranoside, the O-glucose is cleaved first before the occurrences of these sugar cleavages. These C-sugar cleavages were not observed in the xylose moiety in isoshaftoside. These three types of C-sugar moiety cleavage were also observed in mangiferin (Supplement Figure S7), neomangiferin, and aloesin, in which the skeletons of the aglycones are different from other compounds, suggesting that these sugar moiety cleavages are not affected by the skeleton of the aglycone.

#### 2.2.3. Aglycone Cleavage

*B ring loss and C-ring cleavage*: The MS fragmentation pathway of flavonoids has been well-studied, with major fragmentation typically involving cleavages in the C-ring, such as C-ring RDA reaction and alpha reaction. However, in these flavonoid C-glycosides, C-ring cleavage is not observed. Instead, in isovitexin, homoorientin, and swertisin, there is a B-ring loss followed by the loss of a H_2_O to afford fragment ions at *m*/*z* 323.0761 (21%), 339.0711 (19%), and 337.1075 (17%), respectively (Table 2). Additionally, in swertisin, the fragment of the B-ring loss followed by a −CH_2_ loss from the methoxy at position C7 of the aglycone is observed at *m*/*z* 323.0761 with an abundance of 22%. Isoshaftoside also has B-ring loss fragments at *m*/*z* 307.0607 (34%) and 337.0716 (43%) after losing the glucose or xylose moiety, respectively (Table 2). These findings suggest that due to the presence of a C-glycosidic sugar moiety, the fragmentation pathway of the aglycone in C-glycosides is different from those without sugar moieties (i.e., flavonoids).

**Figure 3:**
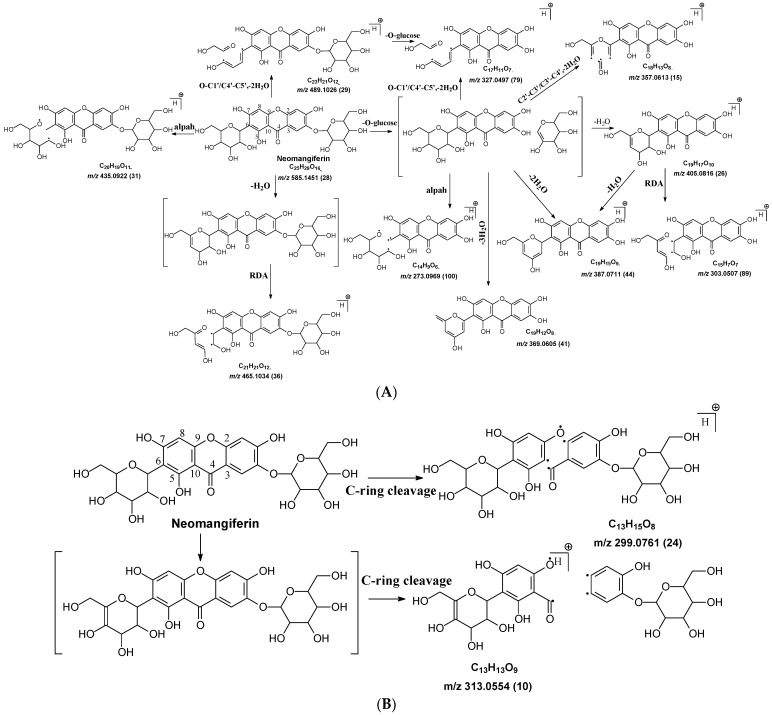

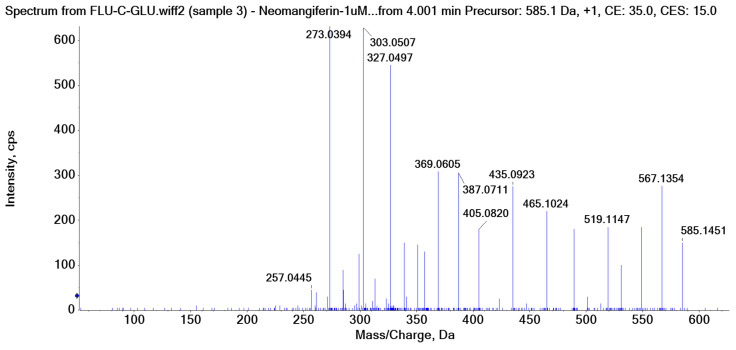
Fragmentation pathway of neomangiferin. (**A**), major sugar moieties fragmentation pathways; (**B**), aglycone fragmentation pathways.

In mangiferin and neomangiferin, which have xanthone as the skeleton of the aglycone, C-ring cleavage is a common fragment pathway. After C ring cleavage, the fragments containing the sugar moiety were observed at *m*/*z* 299.0761, 285.0605, 313.0554 at low or medium abundance (Table 2, Figure 3).

**Figure 4:**
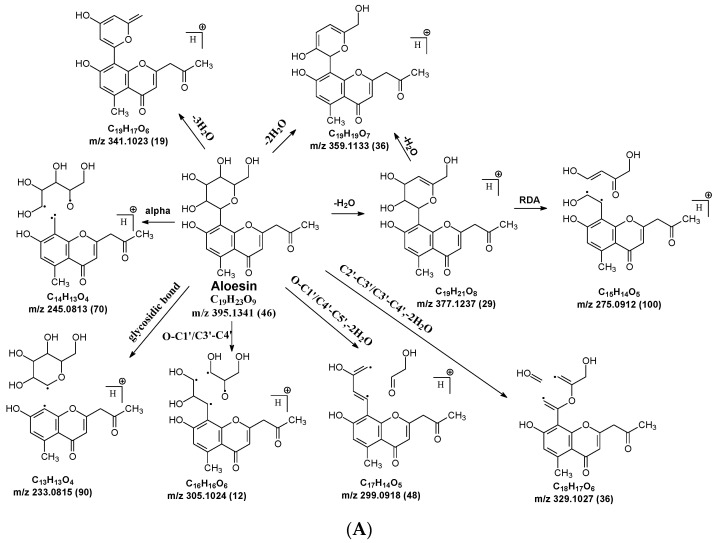

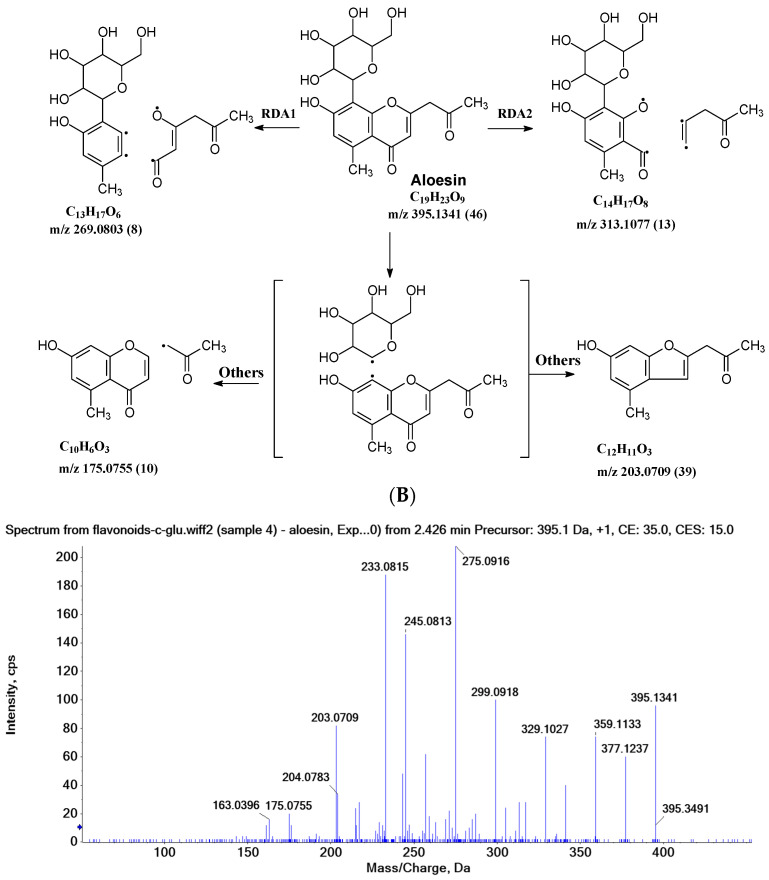
Fragmentation pathway of aloesin. (**A**), major sugar moieties fragmentation pathways; (**B**), aglycone fragmentation pathways.

In aloesin, where there is no B ring in the aglycone, two types of C-ring RDA cleavages were observed, resulting in two fragment ions at *m*/*z* 269.0803 and 313.1077 with an abundance of 8% and 13%, respectively. Additionally, the loss of the ketone side chain at C2 after the glycosidic sugar loss was observed to afford a fragment at *m*/*z* 175.0755 with an abundance of 10% (Figure 4B). These findings further indicated that C-glycoside on the A ring facilitated the loss of the substitute (i.e., B ring or ketone chain) at C2 in the aglycone, as observed in the abovementioned flavonoids C-glycosides.

### 2.3. MS Fragmentation Pathway of Representative Compounds

The common MS fragmentation pathways are presented above. Here, we provide a detailed characterization of the MS fragmentation pathway of three representative compounds, including isoshaftoside, neomangiferin, and aloesin. Isoshaftoside has a flavonoid aglycone with two different C-glycoside sugar moieties attached at position-6 and position-8. Aloesin has an aglycone that is similar to a flavonoid but without the B-ring. Neomangiferin has a different aglycone skeleton from the other two compounds. The aim is to identify and characterize key fragments and elucidate the underlying mechanisms of fragment formation to gain insights into the structural features and functional groups that influence fragment behavior.

#### 2.3.1. Isoshaftoside MS Fragmentation Pathway

Water loss, followed by sugar RDA cleavage, is one of the major fragmentation pathways for isoshaftoside. Isoshaftoside has a C-glucose and a C-xylose moiety attached to the C6 and C8 positions on the flavonoid aglycone. Water loss from the C-glucose or C-xylose affords a fragment at *m*/*z* 544.1444 with an abundance of 38%. Then, a sugar RDA reaction occurs in the glucose and xylose moiety to afford two fragment ions at *m*/*z* 475.1035 (15%, RDA1) and 445.1131 (21%, RDA2), respectively (Figure 2A). Additionally, a fragment with two water loss from these two sugar moieties on isoshaftoside was also observed at *m*/*z* 529.1357 (34%), which underwent further RDA cleavage (RDA3) in the glucose moiety to afford a fragment at *m*/*z* 457.1131 (30%). The fragment from RDA3 further lost one or two H_2_O, resulting in two fragments ion at *m*/*z* 439.1029 (19%) and 421.0927 (25%), respectively (Figure 2A). Interestingly, after this, the fragment from RDA1 underwent further alpha cleavage to afford a fragment ion at 325.0715 (71%), suggesting that different fragmentation pathways can occur sequentially.

Alpha cleavage is another major fragmentation pathway for isoshaftoside. Both the C-glucose and C-xylose can initiate alpha cleavage to afford fragments at *m*/*z* 445.1131 (21%) and 415.1022 (11%), respectively (Figure 2B). Interestingly, other than direct sugar alpha cleavage, the fragment from double water loss (i.e., *m*/*z* 52.01357) undergoes alpha cleavage in the glucose and xylose, resulting in two fragment ions at *m*/*z* 397.0933 (51%) and 427.1004 (26%), respectively. Moreover, these two fragments further underwent alpha cleavage at the xylose or glucose moieties to afford the same fragment ion at *m*/*z* 295.0601 (42%). The fragment from the glucose alpha cleavage after double water loss (i.e., 397.0933) further lost a water unit, resulting in a base fragment ion at *m*/*z* 379.0814 (100%, Figure 2B).

Both C-glycosidic bonded glucose and C-glycosidic bonded xylose can be cleaved directly to release fragments at *m*/*z* 433.0923 and 403.0823 with abundances of 19% and 32%, respectively (Figure 2C). These sugar loss fragments could be further dehydrogenated followed by a B ring loss in the aglycone to afford fragments at *m*/*z* 337.0716 and 307.0607 with abundances of 43% and 34%, respectively. The relatively high abundance of these fragments indicated that C-C Glycosidic bond cleavage followed by B ring loss is one of the major fragmentation pathways for isoshaftoside.

Other sugar moiety cleavages primarily occur in the glucose moiety to afford a fragment ion at *m*/*z* 499.1239 (32%), 469.1136 (38%), and 475.1036 (15%) from C2″–C3″ and C3″–C4″ cleavage, followed by the loss of two H_2_O, and O–C1″ and C4″–C5″ cleavage followed by the loss of two water, and O–C1″ and C3″–C4″ cleavage, respectively (Figure 2D). The similar cleavage in the xylose moiety is not obvious.

#### 2.3.2. Neomangiferin MS Fragmentation Pathway

There is an O-glucose and a C-glucose moiety in the structure of neomangiferin, and the loss of the O-glucose is a fragmentation pathway. However, the fragment ion from losing O-glucose is an intermediate fragment that is further broken into a fragment from the alpha cleavage in the C-glucose moiety at *m*/*z* 273.0969 as the base peak (100%, Figure 3A). Moreover, this O-glucose-losing fragment loses one, two, or three H_2_O from the C-glucose moiety to afford fragment ions at *m*/*z* 405.0816, 387.0711, and 369.0605, with an abundance of 26%, 44%, and 41%, respectively. Furthermore, the fragment with one H_2_O loss at *m*/*z* 405.0816 undergoes RDA cleavage to afford a fragment ion at *m*/*z* 303.0507 with an abundance of 89%. The intermediate O-glucose fragment also cleaved at O–C1″ and C4″–C5″ cleavage, followed by the loss of two H_2_O or C2″–C3″ and C3″–C4″ cleavage, followed by the loss of two H_2_O, resulting in two fragment ions at 327.0497 (79%) and 357.0613 (15%), respectively. The C-glycosidic bond can also cleave from the intermediate O-glucose fragment to afford a fragment ion at *m*/*z* 261.0400, but the abundance is only 9%. The abundances of the fragments derived from O-glucose loss range from high to medium, suggesting that O-glucose loss is the major fragmentation pathway; probably the O-C bond is more cleavage-liable in MS/MS analysis.

Interestingly, alpha cleavage in the C-glucose without losing the O-glucose moiety is also observed at *m*/*z* 435.0922 with an abundance of 31%. Additionally, the fragment from C-glucose water loss followed by sugar RDA cleavage is also observed at *m*/*z* 465.1037 with an abundance of 36%. The C-glucose also cleaved from O–C1″ and C4″–C5″ followed by the loss of two H_2_O to afford a fragment ion at *m*/*z* 489.1026 (29%). These findings suggest that cleavage in C-glucose could occur before the loss of the O-glucose moiety in xanthone-glycosides, but the intensities of these fragment ions range from medium to low.

Another fragmentation pathway is the aglycone cleavage without losing the O-glucose and the C-glucose moieties, indicating the skeleton of an xanthone is more cleavage-liable than a flavonoid, as observed in isoshaftoside. The aglycone fragmentation occurs via RDA cleavage at O–C2 and C3–C4 in the aglycone after a H_2_O loss in the C-glucose moiety to afford a fragment at *m*/*z* 313.0554 with an abundance of 10% and the cleavage at O–C2 and C4–C10 to afford a fragment at 299.0761 with an abundance of 24% (Figure 3B). These fragments were also observed in mangiferin, in which there is only a C-glucose moiety (Table 2). These findings suggested that the aglycone of xanthone is more liable to be broken compared to flavonoids in MS/MS analysis.

#### 2.3.3. Aloesin MS Fragmentation Pathway

The aglycone of aloesin is similar to that of a flavonoid with the absence of the B ring. The major fragmentation pathway for aloesin is the water loss followed by RDA cleavage in the C-glucose moiety as it is in flavonoid C-glycoside, resulting in fragments at *m*/*z* 377.1237 and 275.0912 with an abundance of 29% and 100%, respectively (Figure 4A). Additionally, like the flavonoid glycoside, the fragment of the alpha cleavage in the C-glucose is also observed at *m*/*z* 245.0813 with an abundance of 70%. The C-glucose also cleaved at O–C1′ and C3′–C4′ to form a fragment ion at *m*/*z* 305.1024 (12%), at O–C1′ and C4′–C5′ with the loss of two H_2_O units to form a fragment ion at *m*/*z* 299.0918 (48%), and at C2′–C3′ and C3′–C4′ followed by the loss of two H_2_O to afford a fragment ion at *m*/*z* 329.1027 (36%). These findings indicate that sugar cleavage is the major fragmentation pathway in aloesin, and the sugar cleavage is not affected by the absence of the B-ring when compared to those in flavonoid C-glycoside. Moreover, double and triple water loss directly from the C-glucose of the patent compounds was also observed to afford two fragment ions at *m*/*z* 359.1133 (36%) and 341.1023 (19%). Interestingly, in aloesin, the fragment from the C-C glycosidic bond cleavage at *m*/*z* 233.0815 (90%) is more abundant than those in flavonoid C-glycosides where the abundance is only 32% or lower, indicating that the C-C glycosidic bond in aloesin is more liable to be cleaved.

Unlike flavonoid C-glycoside, the fragments from aglycone cleavage can occur in aloesin. The RDA cleavage in the aglycone without losing the C-glucose is the major fragmentation pathway to afford fragments at *m*/*z* 313.1077 (13%) and 269.0803 (8%, Figure 4B). However, the abundance of these two fragments is low, suggesting that the aglycone C-ring broken without sugar loss is not the major fragmentation pathway. Additionally, when the C-glucose moiety is lost, the side chain on the aglycone can be cleaved to afford a fragment at *m*/*z* 175.0755 (10%), or the carbonyl can be cleaved to afford a fragment at *m*/*z* 203.0709 (39%, Figure 4B).

## 3. Materials and Methods

### 3.1. Chemical Sand Reagents

The standard compounds vitexin, isovitexin, swertisin, homoorientin, isoshaftoside, isoscoparin, isoscoparin-2″-*O*-glucoside, mangiferin, neomangiferin, and aloesin (purity > 98%) were purchased from AvaChem LLC (San Antonio, TX, USA). LC-MS grade acetonitrile, methanol, and water were purchased from VWR (Radnor, PA, USA). Other chemicals were used as received.

### 3.2. Sample Preparation

The stock solution of the 10 C-glycosides was prepared in DMSO at 10 mM. Briefly, 5–10 mg of each compound was weighted accurately using an analytical balance and dissolved in a certain volume of DMSO to form a 10 mM stock solution. The working solution for MS analysis was prepared by diluting 20 μL of the stock solution into a 0.1% formic acid–acetonitrile mixture (50:50%). The working solution of each compound was then centrifuged at 15,000× *g* at 4 °C for 15 min for injection.

### 3.3. Instrumentation and Analytical Conditions

The C-glycosides fragmentation was performed on an UHPLC-X500B QTOF mass spectrometer (SCIEX, Framingham, MA, USA). The separation was achieved on an Acquity UPLC BEH C18 column (50 × 2.1 mm, 1.7 mm; Waters Corporation, Milford, MA, USA). The mobile phases were water containing 0.1% formic acid (A) and acetonitrile (B). The flow rate was set at 0.4 mL/min. The autosampler and column oven temperatures were 15 °C and 30 °C, respectively. The running time was 15 min. The time program of the gradient was as follows: Phase B was initially at 10% for 2 min, increased from 10% to 95% in 10 min, kept at 95% for 1 min, decreased to initial concentration (10%) in 0.5 min, and equilibrated for 1.5 min.

The QTOF MS/MS is equipped with a TurboV ESI ion source, operated in the positive and ion modes. TOF-MS and TOF-MS/MS data were acquired using the Information-dependent-acquisition (IDA) in positive mode. A survey scan, IDA QTOF-MS, was performed to collect the information on the precursor ions, followed by multiple dependent QTOF MS/MS scans for several of the most abundant precursor/candidate ions. The temperature and spray voltage were set at 500 °C and 5000 v, respectively. The curtain gas, collision gas (CAD), nebulizer gas (gas 1), and heater gas (gas 2) were set at 30, 10, 55, and 60 psi, respectively. The QTOF-MS was set over a *m*/*z* range from 100 to 1000 with an accumulation time of 0.2 s. Declustering potential (DP) and collision energy (CE) were 50 v and 10 v, respectively. QTOF-MS/MS was set over a *m*/*z* range from 50 to 1000 with an accumulation time of 0.05 s, and DP and CE were 50 v and 35 v with a 15 v spread, respectively. Data were acquired using SCIEX OS software 1.6.1.

## 4. Conclusions

Glycosides with similar aglycones but different sugar moieties attached at different positions of the aglycone through C-O or C-C bonds have been investigated using UHPLC-QTOF-MS/MS in this paper. By comparing the fragmentation pathways of these analytes, we conclude that the cleavage in the sugar moiety is more prevalent in C-glycosides compared to that in O-glycosides. This is probably because the glycosidic bond in O-glycosides is the most liable bond to be cleaved and loses a neutral sugar moiety in MS/MS analysis is very common. In C-glycoside, the major sugar fragmentation pathway is water loss, followed by RDA and alpha cleavages. We predict that these fragments are the most sensitive transitions in MRM scan for C-glycosides’ quantification. Other cleavages can also occur at positions C2–C3 and C3–C4, O–C1 and C3–C4, and O–C1 and C4–C5 with H_2_O loss in the sugar moiety. We anticipate that these MS/MS fragmentation pathways can be used for C-glycosides’ structure elucidation from diets, as well as for developing quantitative methods using MRM scan in C-glycoside quantification for the quality control of dietary products using triple quadrupole mass spectrometers.

## Figures and Tables

**Table 1:** The structures and major food sources of the 10 C-glycosides.

Compounds	Structure	Major Sources	References
Vitexin	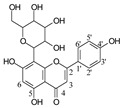	Buckwheat, Hawthorn, Mung beans, Passiflora	[15]
Isovitexin	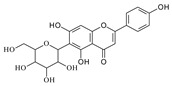	Barley, Cucumber	[15,16,17]
Swertisin	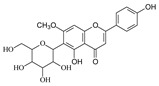	Jujube	[18]
Homoorientin (Isoorientin)	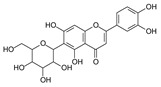	Passion fruit	[19]
Isoshaftoside	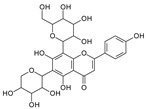	Barley	[16,20]
Isoscoparin	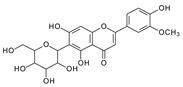	Yellow grain rice	[21]
Isoscoparin-2″-O-glucoside	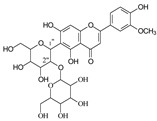	Yellow grain rice	[19,22]
Mangiferin,	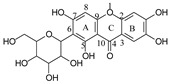	Mango, Coffea, Honeybush	[23,24]
Neomangiferin	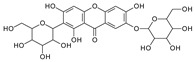	Mango	[24]
Aloesin	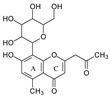	Aloe	[25]

**Table 2:** Major sugar moiety, glycosidic bond, and aglycone fragments of the C-glycosides (*m*/*z*, abundance %).

	Sugar Moiety								Glycosidic Bond
Compounds	-H_2_O (−18)	-2H_2_O (−36)	-3H_2_O (−54)	-H_2_O/RDA (−120 or 90)	Alpha (−150 or 120)	C_2_–C_3_ and C_3_–C_4_, -2H_2_O, (−66)	O–C_1_ and C_3_–C_4_ (−90)	O–C_1_ and C_4_–C_5_ -2H_2_O (−96)	−162 or 132
Isovitexin	415.1024 (11)	397.0918 (19)	379.0816 (21)	313.0707 (86)	283.0601 (100)	367.1024 (14)		337.0711 (45)	271.0601 (8)
Homoorientin	431.0973 (8)	413.0867 (20)		329.0656 (80)	299.0550 (100)	383.0766 (17)		353.0660 (42)	287.0550 (9)
Isoscoparin	445.1136 (16)	427.1024 (21)	409.0925 (21)	343.0814 (76)	313.0712 (100)	397.0930 (19)		367.0820 (45)	301.0717 (6)
Vitexin	415.1024 (39)	397.0918 (23)	379.0820 (8)	313.0707 (39)	283.0601 (16)	367.1024 (10)	343.0812 (7)	337.0725 (9)	
Swertisin	429.1180 (6)	411.1074 (12)	393.0988 (12)	327.0863 (39)	297.0757 (100)	381.1180 (14)		351.0869 (33)	
Isoshaftoside	547.1444 (38)	529.1357 (34)	511.1247 (25)	475.1036 (15), 445.1131 (21) 457.1131 (30, -2H_2_O)	445.1131 (21), 415.1022 (11), 397.0933 (51, -2H_2_O) 427.1004 (26), 295.0601 (42, -2H_2_O) 379.0814 (100, -3H_2_O) 325.0715 (71, RDA+alpha)	499.1239 (32)	475.1036 (15)	469.1136 (38)	403.0823 (32) 433.0932 (19)
Isoscoparin-2′′-O-glucoside	445.1127 (47, -O-glu -H_2_O)	427.1027 (34, -O-glu -2H_2_O)	409.1341 (31, -O-glu)	343.0812 (100, -O-glu,)	313.0707 (65, -O-glu,)			367.0812 (69, -O-glu)	463.1231 (75, -O-glu) 301.0707 (9, -O glu, -C-glu)
Mangiferin	405.0816 (6)	387.0711 (15)	369.0609 (18)	303.0499 (61)	273.0394 (100)	357.0609 (8)		327.0507 (33)	261.0394 (8)
Neomangiferin	567.1354(30) 405.0816 (26, -O-glu, -H_2_O)	549.1233 (23) 387.0711 (44, -O-glu, -2H_2_O)	531.1140 (12) 369.0605 (41, -O-glu)	465.1028 (36) 303.0507 (89)	435.0922 (31); 273.0969 (100, -O-glu)	519.1147 (32) 357.0613 (15)		327.0497 (79, -O-glu) 489.1026 (29)	261.0400 (9), 405.0812 (26, -O-glu-H_2_O)
Aloesin	377.1237 (29)	359.1133 (36)	341.1023 (19)	275.0912 (100)	245.0813 (70)	329.1027 (36)	305.1024 (12)	299.0918 (48)	233.0815 (90)
**Aglycone**									
Compounds	B-ring loss	Loss H_2_O and B ring		Loss sugar and B ring		C-ring cleavage	Others		
Isovitexin		323.0761 (21)							
Homoorientin	339.0711 (19)	311.0761 (11)							
Isoscoparin	339.0867 (19)								
Vitexin									
Swertisin		337.1076 (17)					323.0761 (22, Loss B-ring and CH_3_O) 267.0652 (10, Loss CH_3_O after sugar alpha cleavage)		
Isoshaftoside				307.0607 (34) 337.0716 (43)					
Isoscoparin-2′′-O-glucoside				339.0872 (6)					
Mangiferin						299.0761 (6), 313.0554 (13)			
Neomangiferin						299.0761 (24), 313.0554 (10)			
Aloesin						269.0803 (8), 313.1077 (13)	175.0755 (10), 203.0709 (39)		

## Data Availability

Data are contained within the article and supplementary materials. All the raw data and experimental records are stored in the Research Infrastructure Core (RIC) under the Center for Biomedical and Minority Health Research (CBMHR) at TSU. Raw data are available for research purposes upon request.

## Supplementary Materials

The following supporting information can be downloaded at https://www.mdpi.com/article/10.3390/molecules29132953/s1.
