# Supplementary material for: Fragmentation Patterns of Phenolic C-Glycosides in Mass Spectrometry Analysis
Source: Molecules. 2024 Jun 21;29(13):2953. doi: 10.3390/molecules29132953 (PMC11243344; doi:10.3390/molecules29132953)
Supplement: Supplementary file 1 [file molecules-29-02953-s001.zip › molecules-3047072-supplementary.pdf]

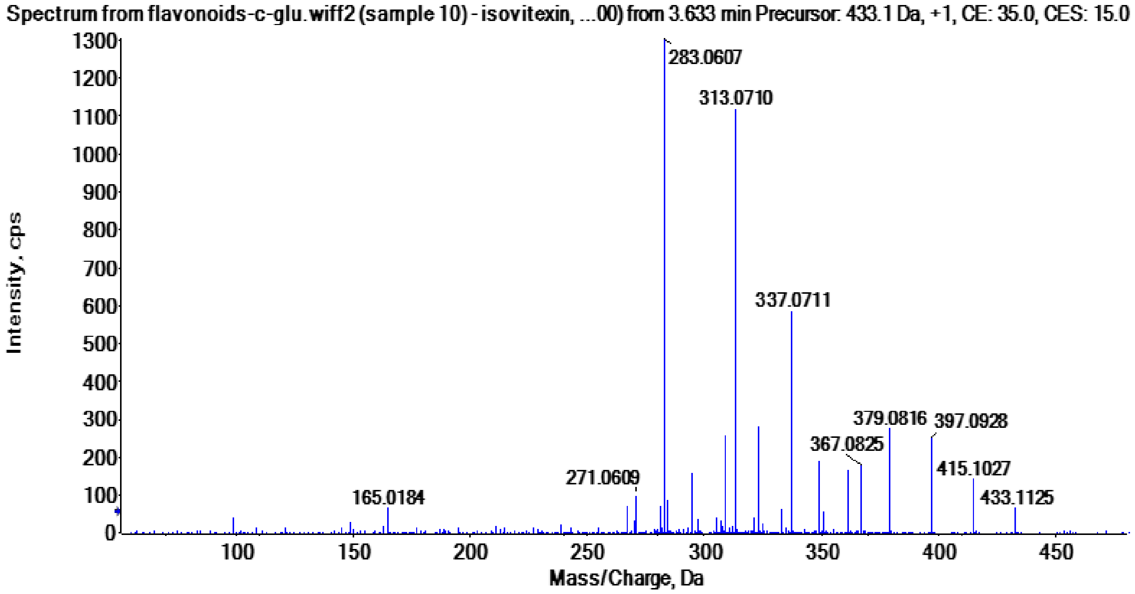

Figure S1: MS/MS spectrum of Isovitexin

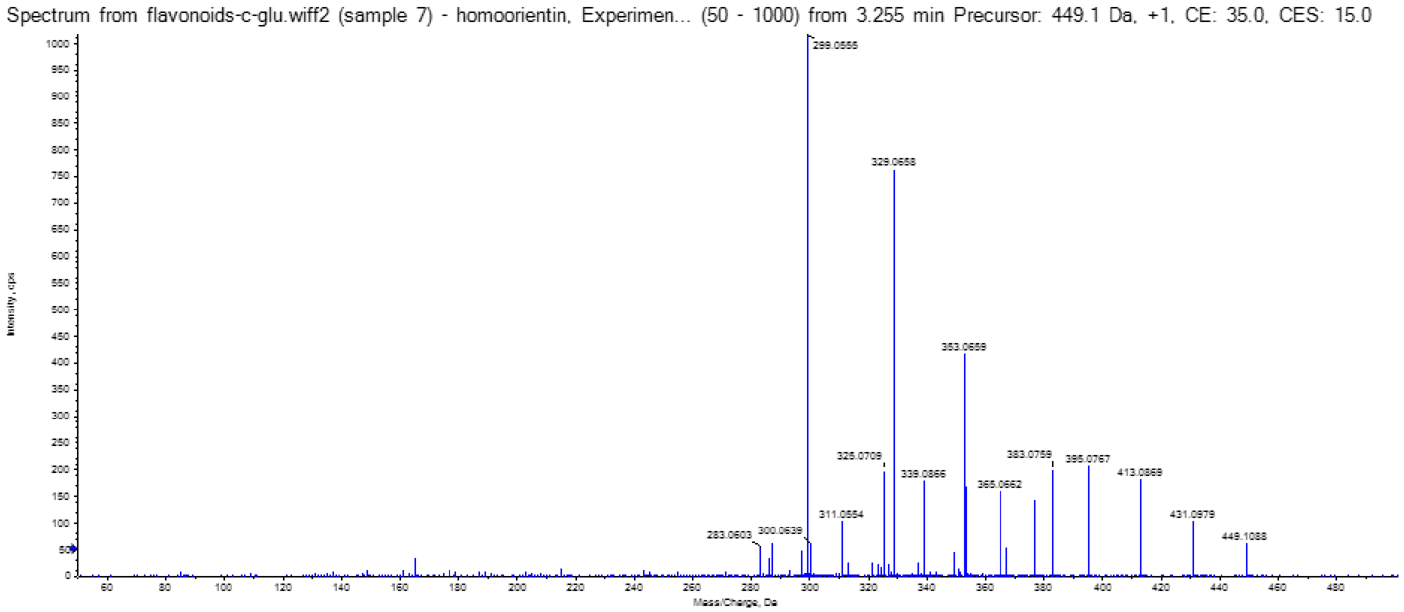

Figure S2: MS/MS spectrum of Homoorientin

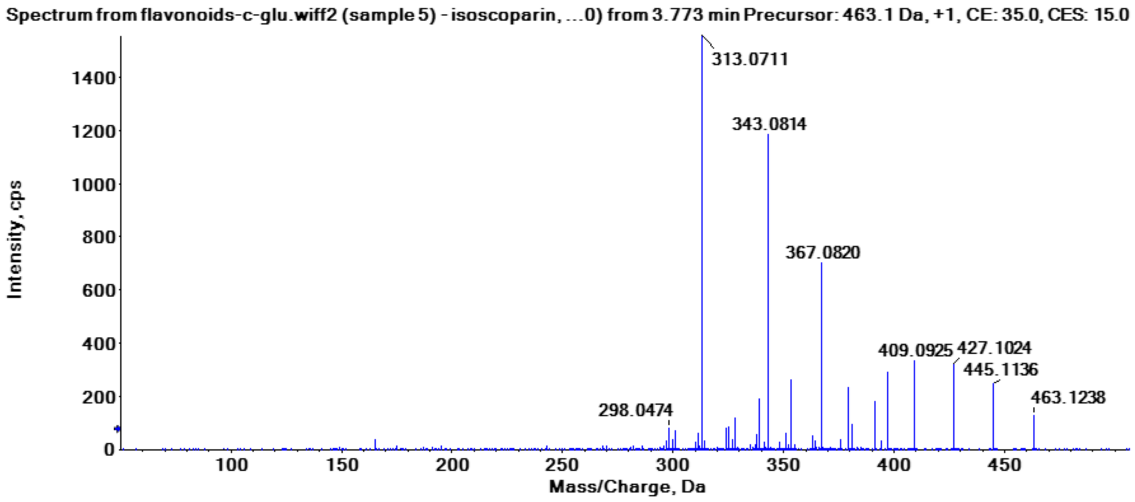

Figure S3: MS/MS spectrum of Isoscoparin

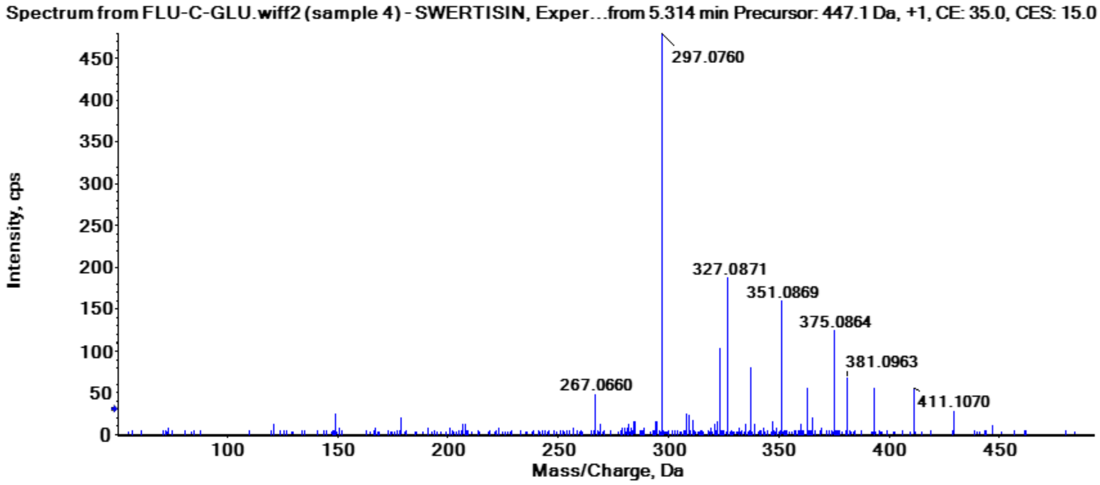

Figure S4: MS/MS spectrum of Swertisin

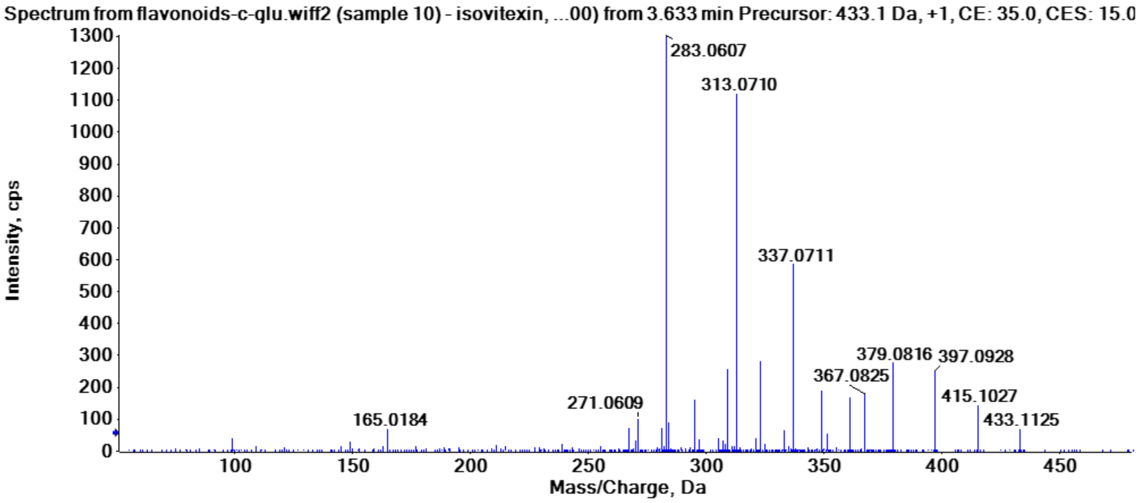

Figure S5: MS/MS spectrum of Vitexin

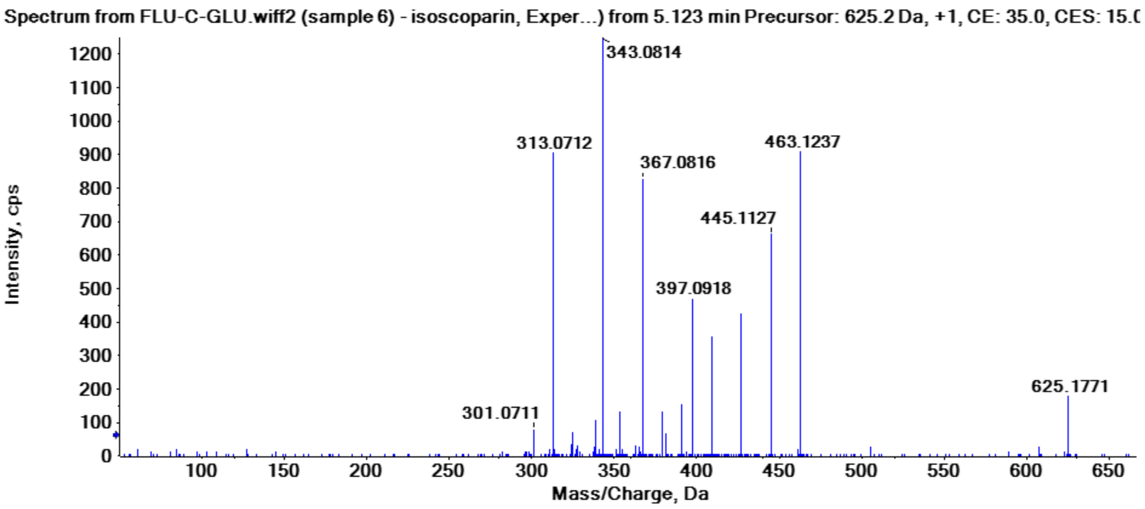

Figure S6: MS/MS spectrum of Isoscoparin-2"-O-glucoside

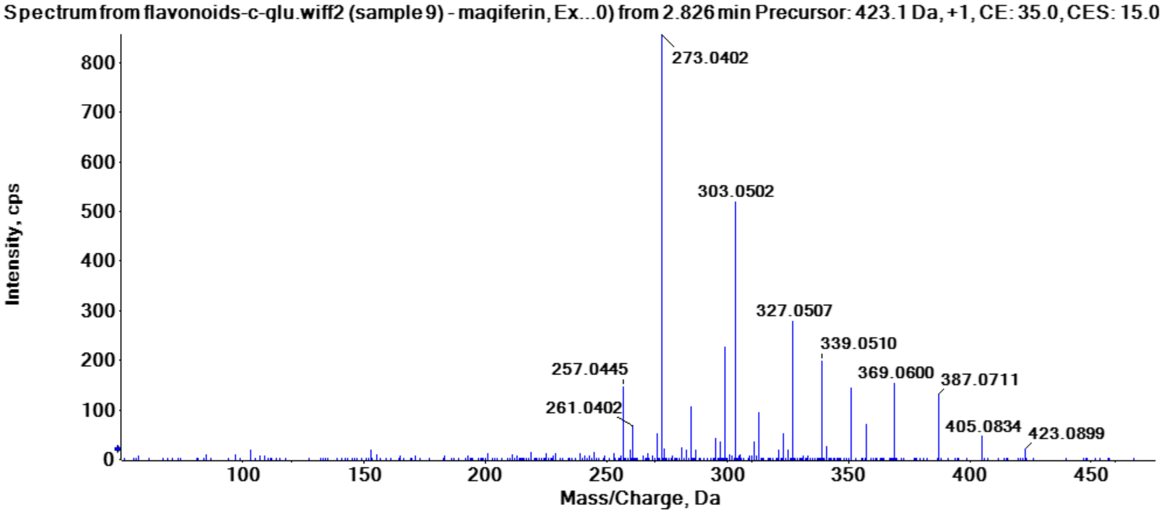

Figure S7: MS/MS spectrum of Mangiferin
